# Topological organization of connectivity strength in the rat connectome

**DOI:** 10.1007/s00429-015-0999-6

**Published:** 2015-02-20

**Authors:** Martijn P. van den Heuvel, Lianne H. Scholtens, Marcel A. de Reus

**Affiliations:** Department of Psychiatry, Brain Center Rudolf Magnus, University Medical Center Utrecht, Heidelberglaan 100, Room: A01.126, 3508 GA PO Box 85500, Utrecht, The Netherlands

**Keywords:** Rat, Connectome, Connectivity, Cortex, Network, Axonal pathways

## Abstract

The mammalian brain is a complex network of anatomically interconnected regions. Animal studies allow for an invasive measurement of the connections of these networks at the macroscale level by means of neuronal tracing of axonal projections, providing a unique opportunity for the formation of detailed ‘connectome maps’. Here we analyzed the macroscale connectome of the rat brain, including detailed information on the macroscale interregional pathways between 67 cortical and subcortical regions as provided by the high-quality, open-access BAMS-II database on rat brain anatomical projections, focusing in particular on the non-uniform distribution of projection strength across pathways. First, network analysis confirmed a small-world, modular and rich club organization of the rat connectome; findings in clear support of previous studies on connectome organization in other mammalian species. More importantly, analyzing network properties of different connection weight classes, we extend previous observations by showing that pathways with different topological roles have significantly different levels of connectivity strength. Among other findings, intramodular connections are shown to display a higher connectivity strength than intermodular connections and hub-to-hub rich club connections are shown to include significantly stronger pathways than connections spanning between peripheral nodes. Furthermore, we show evidence indicating that edges of different weight classes display different topological structures, potentially suggesting varying roles and origins of pathways in the mammalian brain network.

**Electronic supplementary material:**

The online version of this article (doi:10.1007/s00429-015-0999-6) contains supplementary material, which is available to authorized users.

## Introduction

Neural systems include a complex network of structurally and functionally linked elements. Studies examining the architecture of the neural networks of mammalian and non-mammalian species -including the macroscale network of the human, macaque and cat brain, but also the microscale neural systems of lower order nematode species- have shown ample evidence that the wiring diagram of organisms, their ‘connectome’, shows several features of an efficient communication network (Sporns et al. 2005; Bullmore and Sporns 2009; van den Heuvel and Hulshoff Pol 2010a; Sporns 2011). A fundamental attribute of an organism’s connectome appears to be its combined ability to process specialized information and to efficiently integrate neural information across different domains (Sporns 2012). It has been hypothesized that the formation of local densely clustered communities may ensure segregation of information and local specialization, while the presence of global shortcuts may provide an infrastructure for global communication between remote regions (Bullmore and Sporns 2009). Indeed, embracing network science as a theoretical framework to examine the wiring of neural networks, computational studies have consistently shown brain networks to display cost-effective wiring and a small-world modular organization with high levels of local clustering and pronounced modular structure, combined with short communication pathways ensuring efficient global communication (Hagmann et al. 2008; Bassett and Bullmore 2006; van den Heuvel et al. 2008c). An important role in the formation of short communication routes has been suggested to be occupied by a relatively small number of highly connected hub nodes. As a set of densely interlinked regions, hub nodes have been noted to form a central ‘rich club’ or ‘core’, constructing a spatially diffuse, but topologically central system, suggested to be important for global neural communication and thus integration of information between otherwise segregated functional systems (Tomasi and Volkow 2010; van den Heuvel and Sporns 2013b; Cole et al. 2010; de Reus and van den Heuvel 2014).

In humans, most of the connectome work is based on in vivo neuroimaging data, with macroscale pathways and indirect measures of anatomical connectivity strength derived from diffusion-weighted imaging (e.g. Hagmann et al. 2008; van den Heuvel et al. 2008b). In contrast, animal studies allow for a detailed reconstruction of macroscale white matter pathways by means of neural tracing of axonal projections (e.g. Scannell et al. 1995; Goldman-Rakic 1988). By collecting data across a large number of tract-tracing studies, detailed reconstructions of the connectomes of (among other species) the macaque, cat, rat and mouse have been made (e.g. Oh et al. 2014; Scannell et al. 1995; Stephan et al. 2001; Markov et al. 2011; Bota and Swanson 2007), and examinations of the topology of these networks have shown ample evidence of global organizational principles similar to those observed in the human brain (e.g. Sporns et al. 2007; Harriger et al. 2012; Li et al. 2013; Hagmann et al. 2008; Goulas et al. 2014). An advantage of tract-tracing data is that it allows for assessment of the directionality of the brain’s white matter projections, providing important information that is out of the scope of current in vivo imaging-based reconstructions of the human connectome. Moreover, in case of animal connectomes, some reconstructions provide detailed information on the connectivity strength of axonal projections, allowing the examination of the effect of the presence of weaker and stronger pathways on global network properties. Here, analyzing the directed and weighted macroscale connectome reconstruction of the rat brain as provided by the BAMS-II project (Bota and Swanson 2007)—arguably one of the most detailed datasets on mammalian connectome wiring with information on the connectivity of cortical and subcortical regions, including direction, as well as connectivity strength of pathways- we provide new insights into the architectural attributes of the mammalian connectome. We first show (part I) that the rat connectome has similar topological attributes as previously shown for the human, macaque, cat and mouse connectome, including a modular structure, short communication pathways, and a dense central rich club. Next, extending previous observations (part II), we show distinct network organizations of weak versus strong network connections, suggesting varying network roles of edges of different connectivity strength in the rat connectome.

## Materials and methods

### Connectome reconstruction

#### Database and parcellation scheme

Data on macroscale white matter pathways of the rat brain was taken from the open-access BAMS-II connectivity database (http://brancusi1.usc.edu/connectome/; Bota and Swanson 2007), involving a comprehensive dataset of tract-tracing experiments of the nervous system of the rat. The BAMS-II database includes information on white matter pathways between 71 non-overlapping regions covering one hemisphere of the rat cerebrum, including a number of subcortical regions, together providing a relatively detailed parcellation of the rat brain (see Supplemental Table S1 for regions and abbreviations). The BAMS-II database is a highly detailed database of macroscale rat brain connectivity, used and described in several studies (French and Pavlidis 2011; Leergaard et al. 2012; Wolf et al. 2011) and with one of the most recent releases described in detail here (Bota et al. 2012; Bota and Swanson 2007).

#### Binary connectivity

**Fig. 1:**
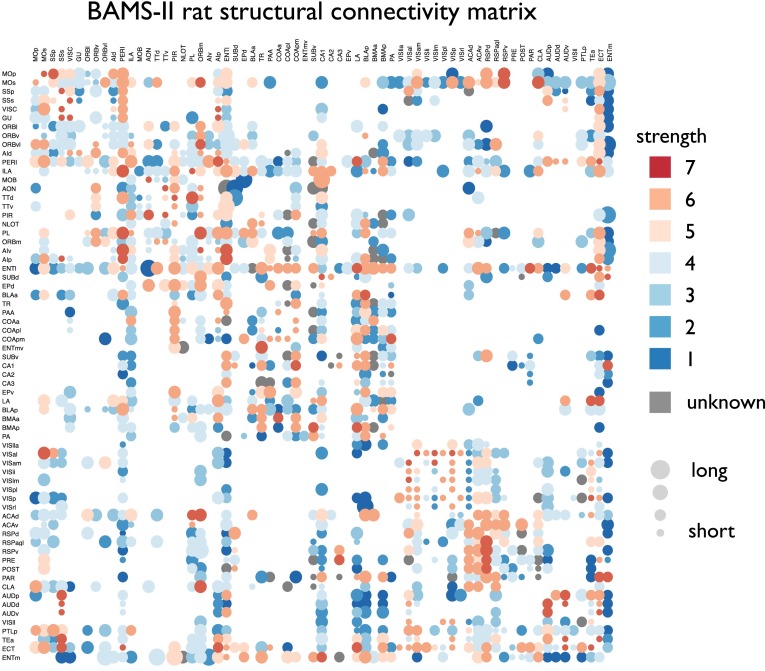
Connectivity matrix of the BAMS-II RAT connectome dataset. Edges reflect the report of an anatomical projection between brain regions, with the level of connectivity strength of edges varying between very weak (edge strength 1) and very strong (edge strength 7)

The BAMS-II database includes information on the presence of 1,424 directed connections between the 71 cortical and subcortical regions, and experimental reports of the absence of an anatomical projection between another 1,955 region pairs. This level of coverage is comparable to previous studies examining connectome reconstructions of the macaque (Modha and Singh 2010) and cat cortex (Scannell et al. 1995). Four regions (AOB (accessory olfactory bulb), DG (hippocampal region, dentate gyrus), IG (hippocampal region, induseum griseum) and FC (hippocampal region, fasciola cinerea)) were found to have no connections or to show only connections amongst each other and were discarded from the graph theoretical analysis. Based on this information, a directed binary adjacency matrix *A* of size *N* × *N* (*N* = 67) was constructed (Fig. 1, Table S1), expressing the presence of an anatomical projection between two regions with a 1 and absent and/or non-reported connections as a 0. This matrix corresponded to a graph *G* = (*V*, *E*) with *V* being the total collection of 67 nodes, reflecting the cortical and subcortical regions, and *E* the collection of 1,397 directed edges reflecting the directed anatomical pathways between regions.

#### Connectivity strength

Besides information on the absence or presence of connection pathways between regions of the rat brain, the BAMS-II database provides detailed information on the strength of the white matter pathways as derived directly from the tract-tracing experiments. Connections are categorized in 7 strength classes, ranging from 1 (very weak) to 7 (very strong). Data on projection strength was available for 1,337 connections (95.7 % of all reported binary connections). In this study, the 64 connections of which the projection strength was unknown were excluded from analyses in which weight information was examined (i.e. in binary analyses these connections were included). Information on the weights of the projections was collected in the directed weighted connectivity matrix *W*, with information on the connectivity strength of a white matter pathway projecting from region *j* to region *i* appearing as entry *W*_ij_. Figure 1 depicts the weighted directed connectivity matrix as derived from the BAMS-II database.

### Graph theoretical analysis

#### Standard network metrics

Graph theoretical analysis of the rat connectome included the examination of the network’s nodal *degrees* and *degree* *distribution,* expressing the number of efferent and afferent connections of brain regions, *clustering coefficient*, indicative of the level of local connectedness of the network, the network’s *characteristic path length*, indicative of the global communication capacity of the network and *betweenness* *centrality*, indicating the importance of a node in simple communication paths in the network. All network metrics were computed on the basis of the binary directed network. Brief descriptions of the examined metrics are given below (more formal descriptions and formulas are given in (Rubinov and Sporns 2010; van den Heuvel and Sporns 2011))

##### Degree

The degree of a node *i* is equal to the total number of connections attached to *i.* In a similar fashion, the in-degree of node *i* is equal to the total number of afferent connections of *i* and the out-degree is equal to the total number of efferent connections of node *i.*

##### Clustering coefficient 

The binary clustering coefficient *C*_*i*_ of node *i* is equal to the ratio between the existing number of connections between neighbors of node *i* and the total number of possible connections between node *i*’s neighbors. The global clustering coefficient *C* was taken to be the average over *C*_*i*_ for all nodes *i* in the network.

##### Path length

The binary path length *L*_*i*_ of node *i* is equal to the average number of edges traversed when traveling from node *i* to all other nodes in the network (i.e. the average of all topological distances between node *i* and node *j*, for all nodes *j* ≠ *i* in the network). The characteristic (i.e. global) path length *L* of the network is the average over *L*_*i*_ for all nodes *i* in the network.

##### Betweenness centrality

The betweenness centrality *B*_*i*_ of node *i* equals the normalized number of times node *i* is passed when walking along the shortest paths between all node pairs in the network, with nodes with the highest *B*_*i*_ being among the most central nodes in the network.

##### Normalized metrics and small-worldness

*C* and *L* were compared to the clustering coefficient *C*_random_ and path length *L*_random_ of a set of randomly wired graphs [1,000 random networks examined, formed by randomly rewiring the edges of the rat connectome while preserving the degree sequence (Maslov and Sneppen 2002)]. The normalized clustering coefficient *γ* is given by the ratio between *C* and *C*_random_, the normalized path length *λ* is given by the ratio between *L* and *L*_random_. A small-world organization is said to be present when the ratio γ/λ, known as the small-world index, exceeds 1.

#### Modular organization

Modular organization of the rat connectome was determined by means of Newman’s modularity algorithm (Newman 2006). The level of modularity *Q* measures whether nodes that appear in the same module are more often connected than in comparable randomly wired networks, with higher levels of *Q* indicating a more modular structure. This modularity algorithm assigns each node to a unique module (Newman 2006).

##### Participation coefficient

The between-module participation coefficient *P*_*i*_ provides information about to what extent the connections of node *i* are evenly distributed across the different modules of the network (Guimera and Nunes Amaral 2005), with nodes with a high *P*_*i*_ reflecting regions with a high intermodular character.

#### Hubs and rich club organization

Hub nodes are nodes that play a central role in the overall network structure (van den Heuvel and Sporns 2013b). Hub nodes were selected on the basis of a cumulative hub-score (van den Heuvel et al. 2010; Bassett et al. 2008) given by the number of times a node scored among the top 33 % of highest ranking nodes on in-degree, out-degree, betweenness centrality and participation coefficient *P*_*i*_, and among the top 33 % of nodes showing the shortest path length *L*_*i*_. The cumulative hub-score could vary from 0 -representing completely non-central nodes- to 5 -representing the most centrally situated nodes in the network. The subset *H* of nodes showing a cumulative hub-score of 4 or more where classified as hub nodes, all other nodes were classified as peripheral non-hub nodes.

Rich club organization of a network describes the phenomenon of the high degree nodes of a network to share a high level of mutual connectivity, being more densely connected amongst each other than expected on the basis of their individual degree alone (Colizza et al. 2006). Previous studies have noted rich club organization for the human (van den Heuvel and Sporns 2011; Grayson et al. 2014), macaque (Harriger et al. 2012), cat (Zamora-Lopez et al. 2009, 2010, 2011; de Reus and van den Heuvel 2013), avian (Shanahan et al. 2013) and mouse brain (van den Heuvel and de Reus 2014), as well as for the neural systems of nematodes (Towlson et al. 2010). A network is said to show a rich club organization if, for a range of degree *k*, the density of connections between the subset of nodes with a degree higher than *k* is higher than in comparable random networks (Colizza et al. 2006). For each level of degree *k*, the subgraph *S*_k_ consisting of *N*_>k_ nodes that displayed a combined in- and out-degree higher than *k* were selected, with the rich club coefficient Φ(*k*) computed as the ratio between the number of connections *E*_>k_ present in subgraph *S*_*k*_ and the total number of possibly occurring connections in *S*_*k*_ (Colizza et al. 2006; van den Heuvel and Sporns 2011). To compensate for the effect of higher degree nodes in randomized networks to also show a higher probability of being connected, Φ(*k*) is typically compared to the average rich club coefficient Φ_random_(*k*) of a set of randomized graphs with the same degree distribution (i.e. with the same number of nodes and connections and the same degree sequence, but now with an otherwise randomized connectivity structure) (Colizza et al. 2006). A set of 1,000 random graphs was formed, randomly rewiring the connections of the rat connectome, while preserving the in- and out-degree of each node *i* in the network (Maslov and Sneppen 2002). The rich club coefficients of these random graphs formed a null-distribution of the level of connectivity appearing among nodes with degree >*k* under the null-hypothesis. Based on this null-distribution, for each level of *k*, Φ(*k*) was assigned a *p* value as the percentage of the null-distribution that exceeded the value of Φ(*k*). A rich club organization can thus be noted when, for a range of *k,* Φ(*k*) significantly exceeds the average random rich club coefficient Φ_random_(*k*), or put differently when the ratio Φ_norm_(*k*) between Φ(*k*) and Φ_random_(*k*) exceeds 1 (Colizza et al. 2006; van den Heuvel and Sporns 2011).

##### Rich club selection

After establishing the existence of a rich club organization, the rich club of the rat connectome was selected as the set of high degree and highly central nodes *H* (see paragraph above). The rich club coefficient Φ(*H*) of this set of hub nodes was computed as the ratio between the number of existing edges and the total number of possible edges between them, which amounts to replacing *S*_>*k*_ by the set of rich club nodes *H*. Similarly, Φ_norm_(*H*) was computed by comparing Φ(*H*) to Φ_random_(*H*) as computed in a set of randomized networks (1,000 random networks examined).

#### Connection classes

##### Intra vs. intermodular character

Connections were categorized according to the position they occupied in the network. A connection was labeled *intramodular* if it connected two nodes that participated in the same module, and *intermodular* if it connected nodes of different modules.

##### Rich club, feeder, local

In addition to this classification, network edges were labeled as *rich club* connections if they interlinked two rich club hub nodes, labeled *feeder*-*in* if they projected from a non-hub to a hub node, *feeder*-*out* if they projected from a hub node to a non-hub node and *local* if they connected two non-hub peripheral nodes.

##### Directionality

A connection projecting from node *i* to node *j* was labeled *bidirectional* if there was also a projection from node *j* to node *i* present, and *unidirectional* when no such connection was present.

#### Edge metrics

To examine the role of different connection classes in the network, the importance of each individual connection for several network metrics was estimated by computing network metrics both before and after the removal of a connection (de Reus et al. 2014; de Reus and van den Heuvel 2014). The percentage of change resulting from the removal of a connection was then taken as an indication of the relevance of that connection for the examined network metrics. Four metrics were considered, being the impact of a connection on (1) the characteristic path length, (2) the global clustering coefficient, (3) the average (i.e., global) *communicability* between all nodes of the network and (4) the *local* *communicability* between the endpoints of the connection. The communicability measure is a weighted sum (computed as the matrix exponent of *A*) that includes paths of all possible lengths between nodes *i* and *j* in the network, assigning higher weight to shorter paths, and is indicative of the level of theoretical network communication between network nodes (Estrada and Hatano 2008; de Reus and van den Heuvel 2014).

##### Edge module diversity

The edge module diversity (EMD) of connection *c* interlinking nodes *i* and *j* was defined as the product *p*_*i*_·*p*_*j*_ (de Reus and van den Heuvel 2013), with module diversity *p*_*i*_ of node *i* representing the fraction of modules that node *i* connected to. As such, high levels of EMD indicate edge *c* to span between two nodes with access to many different types of information. In contrast, a low EMD expresses connection *c* to facilitate information transfer between nodes that process more unimodal information.

#### Topological organization of connectivity strength

To examine the existence of a potential organizational difference between the network’s weak and strong connections, we investigated the topological network organization of each strength class separately, by examining graph characteristics of the subgraph *A* ^w^ = (*V, E* = *w*) with connectivity matrix:

$$A_{\text{ij}}^{\text{w}} = \left\{ {\begin{array}{*{20}c} 1 & {{\text{if}}\,A_{\text{ij}} = w} \\ 0 & {\text{otherwise}} \\ \end{array} } \right.$$

with *A* being the adjacency matrix representing the connections of the rat connectome and *w* ranging between 1 and 7. Although general caution is needed when interpreting subnetworks *A* ^w^ as independent neural networks with metrics of organization now computed and analyzed in isolation of other connections, this type of analysis could provide further insight into the global distribution of connections of a certain connectivity strength across the total network. Examined metrics of organization of the *A* ^w^ subnetworks included (1) global clustering *C* and modularity *Q*, providing information on the potential local organization of a connectivity strength class, and (2) the global efficiency (inverse of the harmonic mean topological distance over all node pairs; used instead of path length to avoid effects of disconnected nodes) (Latora and Marchiori 2001) and diameter (being the maximum topological distance over all node pairs) of *A* ^w^, providing insight into the global connectivity organization of all *A* ^w^.

## Results

In what follows we first describe the results of the analysis of relatively commonly examined network attributes of the mammalian brain, reporting on a small-world, modular and rich club organization (*part I*), followed by a description of the results of the primary analysis of this study examining the organization and distribution of the projection strength of the connections of the rat connectome (*part II*).

### Part I: connectome descriptives

#### Clustering, path length, small-world

**Fig. 2:**
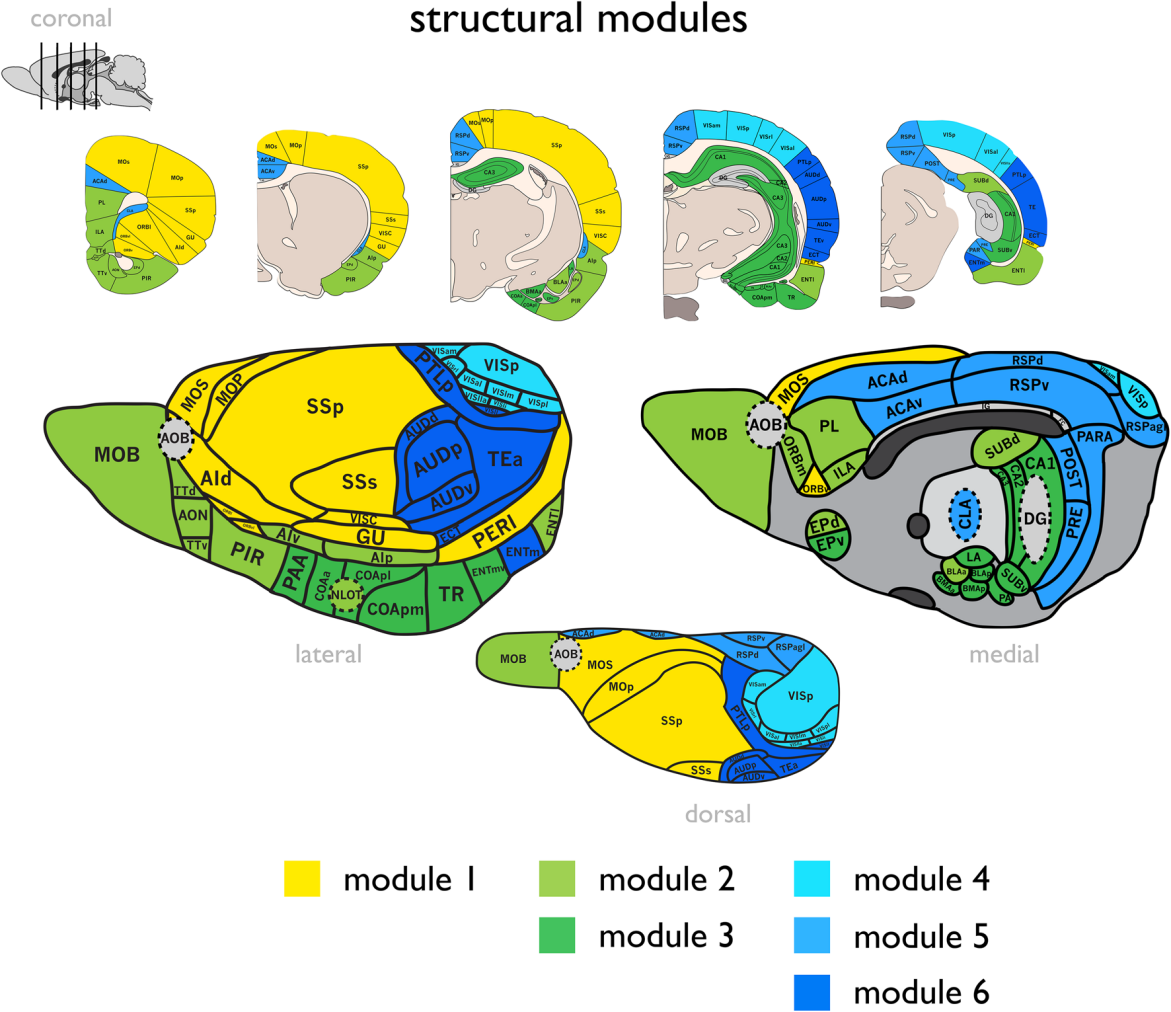
Figure shows the module partitioning of the rat brain in three main modules (*yellow*, *green* and *blue*) and six submodules (respectively 1, 2, and 3 submodules indicated by *color shades* varying from *light* to *dark*). Lateral, medial and dorsal views are artist renderings of figures presented in (Palomero-Gallagher and Zilles 2004); coronal slices are adapted from (Swanson 1992)

Consistent with previous reports on connectome organization of the mammalian brain, the reconstructed rat connectome (Fig. 1) revealed a density of 31.6 %, a right-tailed degree distribution, above random levels of clustering (binary network: 1.18× more than random; weighted network: 1.21× more; 1,000 random networks), a short path length (binary: 1.02× longer than random; weighted: 1.05× longer; 1,000 random networks) and a small-world index larger than 1 (binary: SW = 1.16; weighted: SW = 1.15). The rat connectome revealed a modular structure (binary: *Q* 2.57× higher than in random networks; weighted: *Q* 2.11× higher; 1,000 random networks). Modularity analysis further revealed a hierarchical modular organization with a top split of the network into 3 modules, which themselves consisted of respectively 1, 2 and 3 submodules (Fig. 2).

#### Rich club organization and hubs

**Fig. 3:**
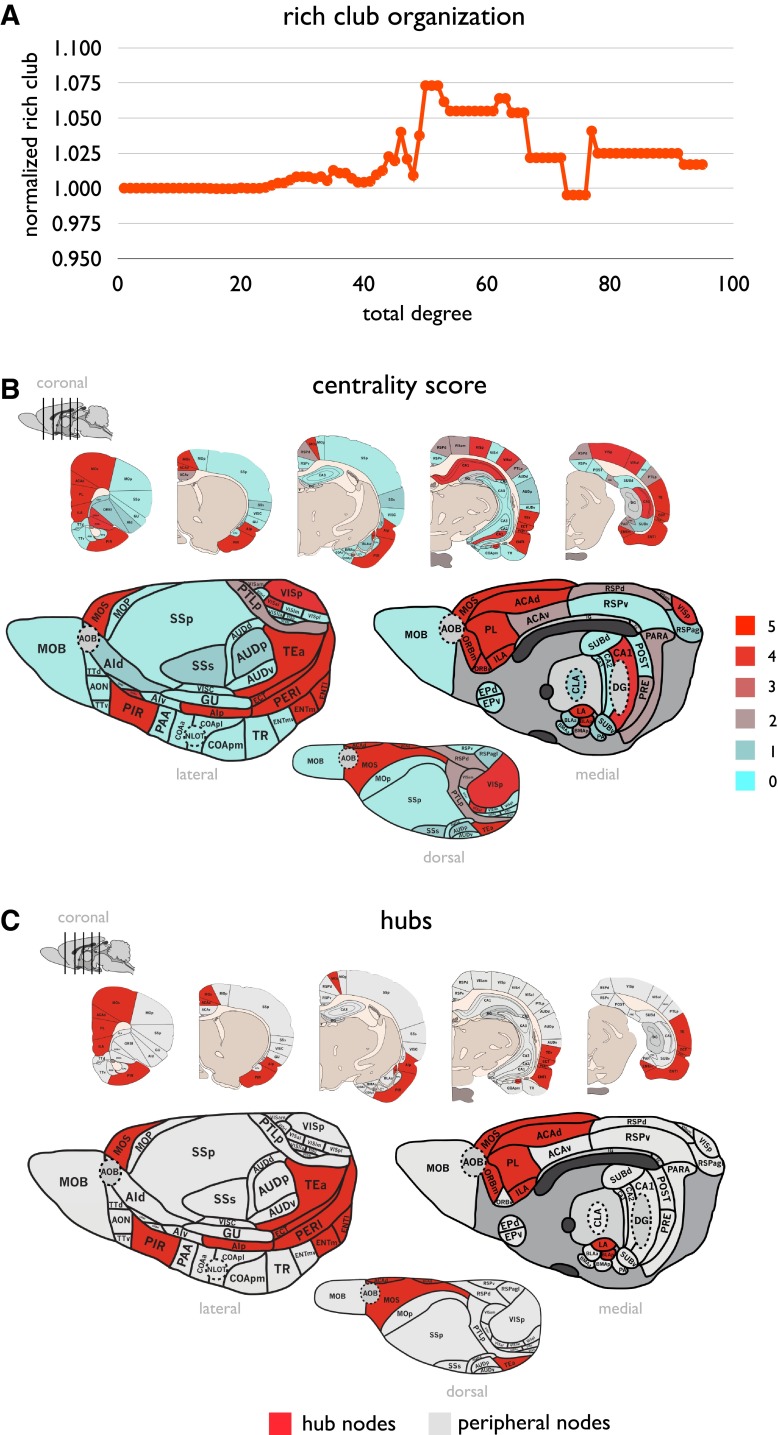
*Panel A* reflects the rich club curve of the rat connectome. The *x*-axis shows the binary number of degree *k* and the *y*-axis shows the normalized rich club coefficient for each set of >*k.* Rich club organization is found to be present for several levels of *k,* as indicated by a normalized rich club coefficient of >1. *Panel B* shows for each brain region the centrality scores ranging from 0 (non-central) to 5 (highly central). *Panel C* hub nodes were taken to be nodes with a centrality score of 4 or more. Hub nodes are depicted in red, non-hub nodes are depicted in gray

The rat connectome revealed a rich club organization. Across the range *50* ≤ *k* ≤ *52*, Φ(*k*) significantly exceeded Φ_random_(*k*) (i.e. Φ_norm_(*k*) > 1, *p* < 0.01, 1,000 random networks examined, Fig. 3a), an observation consistent with the findings of previous reports on rich club organization in other species.

##### Hubs

Hubs taken on the basis of a cumulative hub-score computed as the number of times a node ranked among the 30 % highest ranking nodes on five nodal centrality metrics (see Materials and Methods), revealed the existence of 14 potential hub nodes, all showing a cumulative hub-score of ≥4 (Fig. 3b). This set included regions MOs (secondary motor cortex), ILA (infralimbic area), PIR (piriform area), ACAd (dorsal part of the anterior cingulate area), PL (prelimbic area), ORBm (medial orbital area), AIp (posterior agranular insular cortex), TEa (temporal association areas), ECT (ectorhinal area), PERI (perirhinal area), ENTl (entorhinal area, lateral part), ENTm (entorhinal area, medial part, dorsal zone), LA (lateral amygdalar nucleus) and BLAp (basolateral amygdalar nucleus, posterior part) (Fig. 3c). This set of hub nodes formed a significant rich club with Φ(*H*) being significantly higher than Φ_random_(*H*) (*p* = 0.0010).

#### Rich club and modules

Rich club nodes showed a significantly higher participation coefficient *P*_*i*_ as compared to peripheral nodes (1.20× higher, *p* < 0.001, 10,000 permutations). Moreover, rich club hubs were found to be present in 5 out of 6 2-step modules, underscoring the importance of rich club hub nodes in interlinking modules.

#### Edge statistics

##### Rich club, feeder and local

11 % of the network edges involved rich club connections, 27 % involved feeder-out, 24 % involved feeder-in and 38 % involved local connections.

##### Intermodular vs. intramodular connections

75 % of all rich club connections were intermodular, compared to 72, 76 and 53 % of all feeder-out, feeder-in and local connections respectively. Thus, only 25 % of rich club edges were intramodular connections, in contrast to 47 % of all local connections. Furthermore, rich club connections included 13 % of all intermodular edges of the network, being 1.14× their overall share. Feeder-out connections and feeder-in connections included respectively 29 and 27 % of all intermodular connections (1.09 and 1.13× their overall share). In contrast, local connections were found to be less involved in interlinking different modules (39 %, 0.80× their overall share) and to be more present among intramodular connections (52 %, 1.38× their overall share). Rich club, feeder-out and feeder-in connections included respectively 8, 21 and 17 % of all intramodular connections.

##### Projection distance

Rich club connections spanned significantly longer distances (as estimated by the computed Euclidean distances between the estimated center of mass of all connected region pairs) as compared to local (1.25× longer, *p* < 0.001) and feeder connections (compared to feeder-out: *p* = 0.002; compared to feeder-in: *p* = 0.00246). Furthermore, also feeder-out (1.10×, *p* < 0.001) and feeder-in connections (1.12×, *p* < 0.001) were found to be (on average) longer than local connections.

##### Wiring cost

Rich club connections were found to display a significantly higher cost (computed as physical distance times connectivity strength) than local connections (1.65× higher, *p* < 0.001, 10,000 permutations), as well as than feeder-out (1.36×, *p* < 0.001) and feeder-in (1.22×, *p* = 0.004) connections. These findings underscore an average high wiring cost of rich club edges in the brain network.

##### Directionality

62 % of all connections were found to be bidirectional, 38 % consisted of unidirectional pathways. In addition, 56 % of all intermodular connections were found to be bidirectional (44 % thus being unidirectional). In contrast, the majority of intramodular connections (75 %) were found to be bidirectional (25 % being unidirectional). Rich club connections were found to include predominantly connections of bidirectional nature (94 %, with intermodular connections being 92 % bidirectional and intramodular connections 97 % bidirectional), a percentage significantly higher than expected in random graphs (*p* < 0.001, 1,000 random graphs), with feeder-in (70 %, with intermodular feeder-in connections being 60 % bidirectional) in second place. Feeder-out connections were found to include slightly fewer bidirectional projections (62 %, with intermodular feeder-out connections being 57 % bidirectional), with local projections including 49 % bidirectional projections (with intermodular local connections being just 35 % bidirectional). These findings further converge on the notion of the rich club and rich club edges to form an infrastructure for rich complex neural interactions with an overrepresentation of reciprocal projections (de Reus and van den Heuvel 2013; van den Heuvel and de Reus 2014; Scholtens et al. 2014; Harriger et al. 2012).

##### Edge module diversity

Rich club edges were found to display a higher edge module diversity than local connections (1.26× higher, *p* < 0.001 as compared to the class of local connections, 10,000 permutations), with feeder-out (1.12× *p* < 0.001) and feeder-in edges (1.09×, *p* < 0.001) coming in second and third place.

#### Neural sinks and sources

**Fig. 4:**
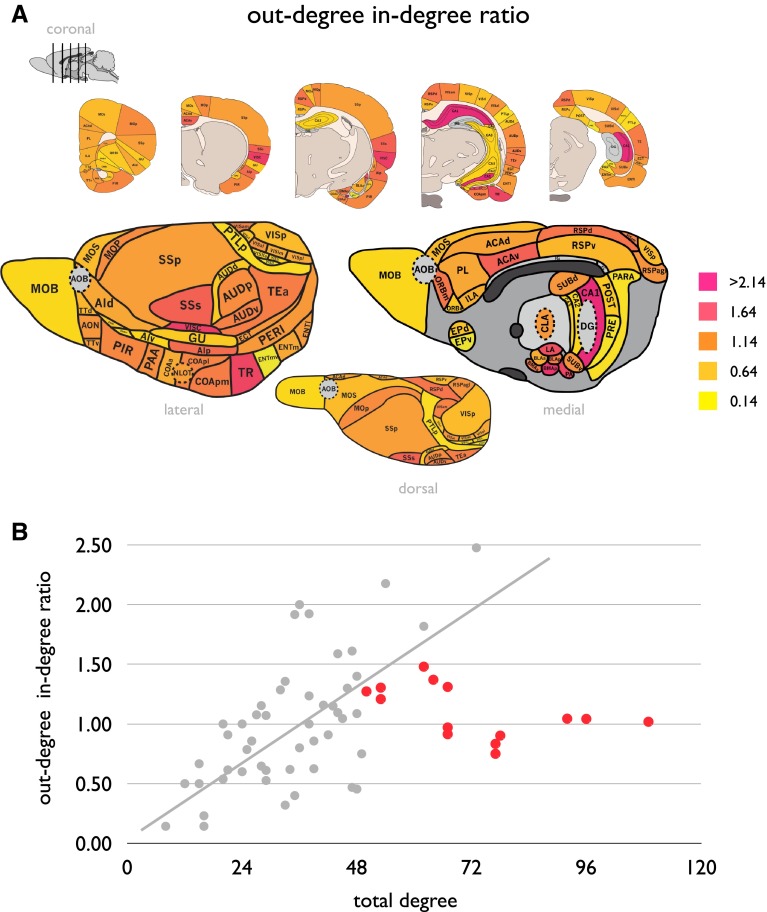
*Panel A* shows the ratio out-degree/in-degree between nodal out-degree and nodal in-degree. Nodes with a low out-degree/in-degree ratio indicate neural sinks (having more incoming connections than having outgoing connections); nodes with a high out-degree/in-degree ratio indicate neural sources, reflecting nodes with more outgoing connections than incoming connections. *Panel B* Out-degree/in-degree ratio correlated significantly with total network degree, Hub nodes (*red points*) appear to be an outlier to this relationship

The ratio between nodal out-degree and in-degree displayed a right-tailed distribution (Fig. 4a), with the majority of nodes showing a ratio around ~1 (73 % between 0.5 and 1.5), suggesting a relative overall balance in the number of efferent and afferent connections of cortical regions. Only a small set of nodes revealed an imbalance, showing more afferent than efferent connections (thus forming “neural sinks”, ratio <0.5, regions: VISll (laterolateral visual area), ORBvl (ventrolateral orbital area), PTLp (posterior parietal association areas), ENTmv (entorhinal area, medial part, ventral zone), PRE (presubiculum), PAR (parasubiculum), EPv (endopiriform nucleus, ventral part), yellow colored regions in Fig. 4a) or more efferent than afferent connections (thus forming “neural sources”, ratio >1.5, regions: SSs (secondary somatosensory cortex), VISC (visceral area), TR (postpiriform transition area), ACAv (ventral part of the anterior cingulte area), CA1 (hippocampal region CA1), LA (lateral amygdalar nucleus), BMAp (basomedial amygdalar nucleus, posterior part), PA (posterior amygdalar nucleus), magenta colored regions in Fig. 4a).

##### Correlation to total degree

Out-degree/in-degree ratio was positively correlated to total nodal degree of a region (Fig. 4b), indicating that regions with a higher total number of pathways on average show a higher number of efferent connections than afferent connections (linear regression, *r* = 0.40, *p* = 0.007).

Hub nodes are noted to form potential outliers to this organizational rule (red nodes in Fig. 4b), showing a relatively balanced number of afferent and efferent connections (out-degree/in-degree ratio mean/std: 1.14/0.29, not different than the ratio of peripheral nodes, *p* = 0.22, 10,000 permutations), likely due to the strong bidirectional character of most of their pathways.

### Part II: topological organization of connectivity strength

#### Relationship between network role of edges and connectivity strength

##### Intermodular vs. intramodular

Intramodular connections involved significantly higher connectivity weights (mean/std: 4.57/1.58) than intermodular connections (3.87/1.66, *p* = 0.002, 10,000 permutations).

##### Rich club vs. feeder vs. local

**Fig. 5:**
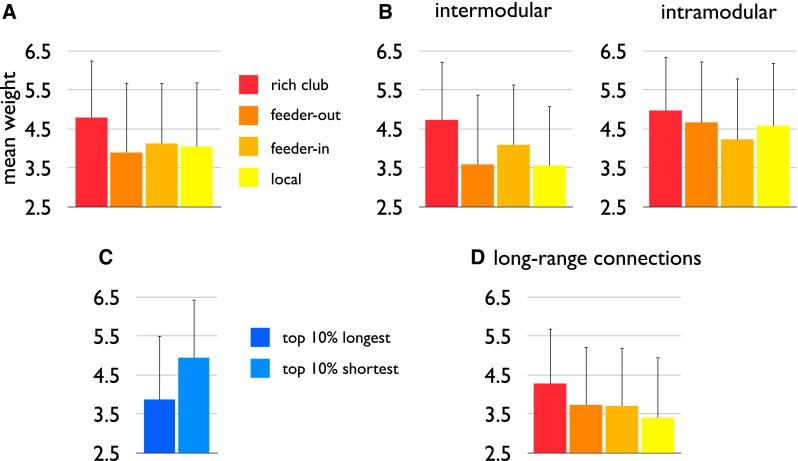
*Panel A* illustrates the average high connectivity weight of the class of rich club connections (*red*) as compared to feeder (feeder-out: *dark orange*, feeder-in: *light orange*) and local edges (*yellow*). *Panel B* illustrates a further subdivision of the class of rich club, feeder and local network edges in intermodular and intramodular connections, showing that rich club edges display the highest connectivity strength of all connections in both classes. *Panel C* shows the average edge weights of the 10 % longest (*dark blue*) and 10 % shortest (*light blue*) connections (as estimated on the basis of Euclidean distance) of the rat connectome. Consistent with previous observations, short connections have a higher average connectivity strength than long connections. *Panel D* illustrates that of the top 10 % longest network connections, rich club connections show the highest connectivity weight, higher than both feeder and local edges

The class of rich club connections was found to show a higher average connectivity strength than the other classes, 1.18× higher than the class of local (*p* < 0.001), 1.23× higher than the class of feeder-out (*p* < 0.001) and 1.16× higher than the class of feeder-in connections (*p* < 0.001). Figure 5a shows the strength distributions of the class of rich club, feeder-out, feeder-in and local connections.

Further categorizing the connections of the network in intermodular and intramodular connections (Fig. 5b), intermodular rich club connections were found to display a significantly higher connectivity strength (mean/std: 4.73/1.50) as compared to the class of intermodular feeder-out (3.3.59/1.79, *p* < 0.001), feeder-in (4.09/1.55, *p* = 0.002) and local connections (3.56/1.52, *p* < 0.001, 10,000 permutations). Moreover, of all strong connections (i.e. weight ≥6) 16 % was found to involve a rich club connection, being 1.46× their overall share, with in contrast 34 % involving local connections (0.89× their share). Furthermore, of all strong intermodular connections (i.e. connections of weight ≥6 spanning between modules), 22 % involved rich club connections (1.96× their overall share), in contrast to only 19 % local connections (0.49× their overall share). For the class of very strong intermodular connections (i.e. weight of 7) these proportions were even bigger, with 40 % being intermodular rich club connections (3.47×) and only 14 % involving local edges (0.37× their share).

##### Short vs. long connections

Short connections (i.e. bottom 10 % of all connections) were found to show stronger projection strengths (mean/std: 4.95/1.64) than long connections (top 10 % of all connections, mean/std: 3.88/1.50, *p* < 0.001, Fig. 5c). Furthermore, also a general correlation between projection length and connectivity strength (*r* = −0.21, *p* < 0.001, linear regression) was observed.

Relating to the three different classes of rich club, feeder and local connections (Fig. 5d), exclusively looking at the subset of the top 10 % longest connections, rich club edges were found to display a trend-level higher connectivity strength (mean/std: 4.29/1.41) as compared to local edges (mean/std: 3.41/1.56, *p* = 0.0318) [similar results were found when selecting the top 15 % or top 20 %].

#### Connectivity strength and topological role of nodes

##### Degree

Examining per network node (i.e. brain region) its ratio HL of high strength (>4) versus low strength (≤4) connections (HL ratio) revealed a significant association between HL and a node’s binary degree (*p* = 0.0056, *r* = 0.33), suggesting that nodes with a high binary high degree (i.e. nodes that display a high number of network edges) show on average more high strength connections than low strength connections (and nodes with a low degree show more low strength edges than high strength edges). Also the inverse of the average path length *L* of a node (computed on the basis of the binary network) correlated significantly with the HL ratio (*p* = 0.0357, *r* = 0.26).

#### Topological organization of connectivity strength classes

**Fig. 6:**
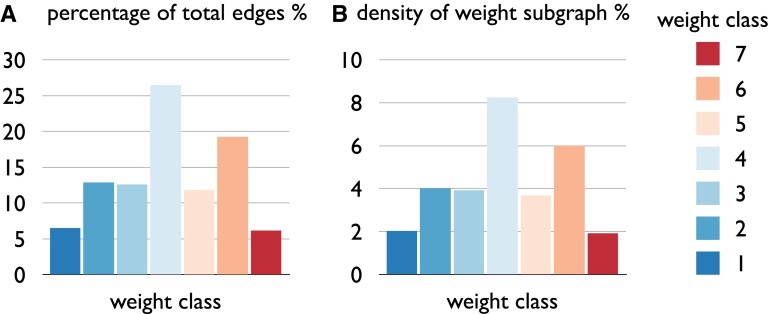
*Panel A* shows the percentage of the total number of edges in the rat connectome dataset belonging to weight class 1 (very weak connections) to 7 (very strong). Percentage of edges per class varied between 6 % (class 1 and 7) and 26 % (class 4). Density values (*panel B*) for each of the selected connection weight subgraphs varied between 2 % (very weak and very strong) and 8 % (class 4)

**Fig. 7:**
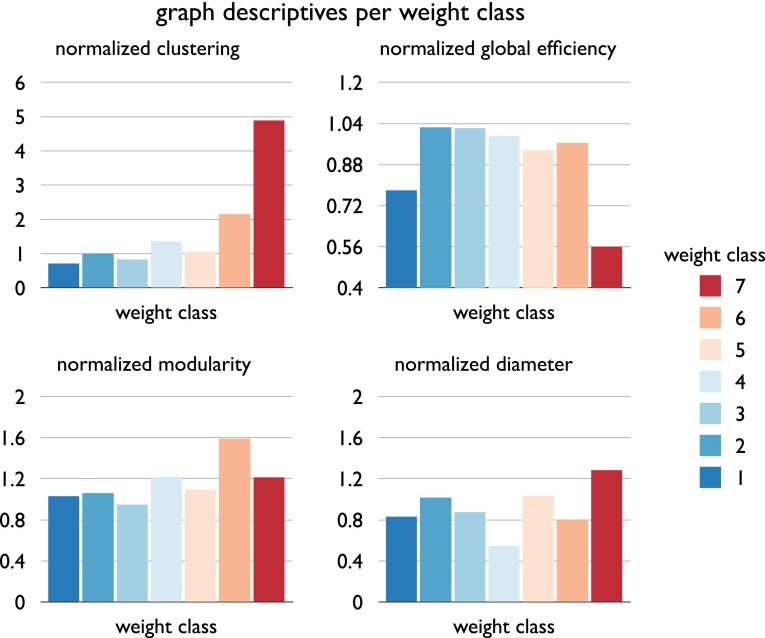
Figure shows the values of descriptive graph metrics computed per weight class. For each weight class, graph metrics were computed for the binary subnetwork of edges. Panels show for each of the seven weight classes (depicted in *different colors* corresponding to Fig. 1) the normalized clustering (as compared to 1,000 randomized networks), normalized path length, normalized modularity and normalized diameter. Figure illustrates different topological structures for different weight classes

Next, we examined the topological organization of the connections within each weight class. The percentage of edges included in each strength class (ranging from 1 (very weak) to 7 (very strong)) is shown in Fig. 6, with the subgraph of very weak (6.5 % of total number of connections) and very strong edges (6.2 %) including the smallest group of edges, and edges of moderate strength including the largest set (26.4 %) (Fig. 6a). As a result, density of the selected weight subgraphs varied between 2 % and 8 % (Fig. 6b). The different subgraphs were found to show distinct topological network features. First, examining metrics of modular organization and clustering, weak edges (strength 1–2) were found to show low levels of normalized clustering *C* (strength 1: 0.8, strength 2: 1.00, normalized to 1,000 random networks) and modularity *Q* (raw 0.37, 0.39; 1.04, 1.10 when normalized to random networks), indicating relatively low local connectivity (Fig. 7). This in contrast to the strong edges of the network (strength 6–7), which show high levels of normalized clustering *C* (strength 6: 2.1, strength 7: 5.3, normalized to 1,000 random networks) and high modularity *Q* (1.59×, 1.21× higher than random). Further examining metrics of global organization, the subgraphs of weak and moderate/strong edges (in particular class 2–6) revealed relatively short communication paths (global efficiency 0.86 to 0.94× that of randomized networks of equal density and equal degree sequence, reflecting a relatively globally oriented organization), while the subgraph of very strong connections showed relatively long communication paths (global efficiency 0.55× that of randomized networks, reflecting a more local and less global organized subnetwork). Consistent with this observation, the subgraph diameter (reflecting the longest path length present, ignoring non-reachable nodes) was found to be relatively small for very weak and weak connections (<1× random level) and relatively large (1.28x random level) for very strong connections (Fig. 7).

#### Edge perturbation

**Fig. 8:**
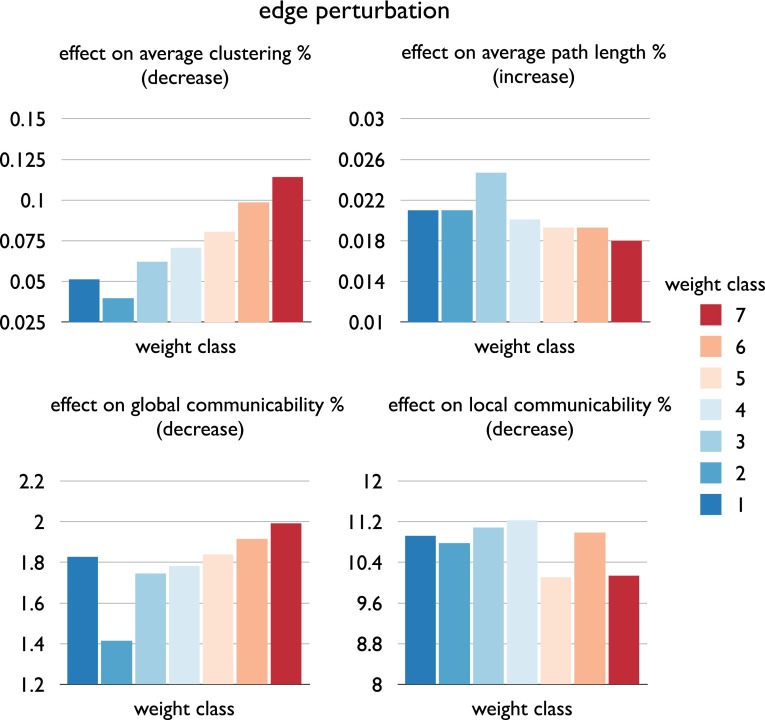
Figure summarizes edge removal statistics for each of the connectivity weight classes. Figure shows the effect of removing an edge of each class (as tested by removing each edge one-by-one and evaluating the effect on the graph metric of interest, with class values reflecting the average over all edges in a class) on binary global clustering, global path length, global communicability and local communicability. Effects are presented as percentage of change (resulting from removal) with respect to the values observed in the original non-damaged network. Note that effects on path length are positive (i.e. reflecting an increase in path length after removal of an edge), while effects on clustering, global and local communicability are negative (i.e. reflecting a decrease in metric after removal of an edge). Note that all metrics were computed on the binary rat network (i.e. with reported effects reflecting the difference between before and after the removal of a single connection of a specific weight class, averaged over all connections in each class), suggesting that the reported effects are related to the impact on the topological organization of the network, with the weight of edges only used to categorize network connections into the different connection classes

Further underscoring a topologically different role in the network of edges of different weight classes, individual (i.e. 1-by-1) removal of strong edges (weight ≥6) was found to have a significantly higher impact (1.16× more) on in particular binary global communicability as compared to the individual removal of weak connections (weight 2–3) (*p* = 0.0014, 10,000 permutations, Fig. 8). Interestingly, weight class 1 appeared to be an outlier to this relationship, suggesting a rather random layout of these connections. Dividing the connections in intermodular vs. intramodular connections, the impact on global communicability was found to be higher for intermodular connections as compared to intramodular connections (1.10, *p* < 0.001, 10,000 permutations. Furthermore, removal of a rich club connection had on average a 4.76× stronger effect on reducing binary global communicability than removal of a local edge in the network, findings consistent with those observed in the human connectome (de Reus and van den Heuvel 2014), underscoring the importance of rich club connections in global network communication (van den Heuvel et al. 2012). Moreover, removal of an intermodular rich club connection had on average a 5.30× stronger impact than removal of an intermodular local edge on global communication. Consistent with previous investigations on edge perturbations (de Reus and van den Heuvel 2014), less pronounced effects were observed on characteristic path length. Notably, the strongest increases in path length were found in the lower weight classes (class 1–3), underscoring a relatively random distribution of these edges, acting as randomly placed global shortcuts in the overall network.

## Discussion

Our findings corroborate on the notion of an organized topological architecture of the macroscale mammalian connectome. Examining a detailed anatomical wiring diagram of the rat brain including a high parcellation of the cerebrum and subcortical nuclei (Bota and Swanson 2007) revealed several topological organizational features of neural network architecture. Supporting findings of previous studies on macroscale connectome organization of other mammalian and non-mammalian species, the rat brain network showed a small-world hierarchical network organization, together with the existence of a small number of high degree, and high strength connected hub regions. Extending previous observations of mammalian connectome organization, our findings now reveal a potential topological organization of connectivity strength across network connections.

First, concerning general descriptives of neural network organization our findings report on several attributes of an efficient processing and communication architecture of the rat brain. Consistent with previous observations on structural and functional connectivity in the rat brain (Liang et al. 2011; Schmitt et al. 2012) and with observations in other mammalian species (e.g. (Salvador et al. 2005; Hagmann et al. 2008; Kaiser and Hilgetag 2006; Stephan et al. 2000; Chatterjee and Sinha 2008; Sporns et al. 2007; van den Heuvel and de Reus 2014), the examined rat connectome showed above random levels of clustering and community structure, indicating the formation of anatomical communities that show overlap with known functional domains in the rat brain (e.g. visual, motor, auditory, frontal networks). In addition to the formation of clustered communities, the network showed the existence of relatively short communication pathways with node pairs from different communities no further apart than three consecutive steps, together indicative of an efficient small-world modular organization. Moreover, again confirming several previous observations, the rat neural network revealed the existence of a set of high degree, highly central hub nodes forming a densely connected rich club (e.g. van den Heuvel and Sporns 2011; Sporns et al. 2007; Towlson et al. 2010; Harriger et al. 2012; Collin et al. 2013; Grayson et al. 2014; Zamora-Lopez et al. 2009). Rich club hub nodes were found to be highly spatially distributed across the brain and to include cortical areas in the far majority of observed communities (Grayson et al. 2014; de Reus and van den Heuvel 2013; van den Heuvel and Sporns 2013a). Validating previous observations in the human connectome -based on in vivo diffusion weighted imaging- connections spanning between rich club nodes revealed several distinct properties as compared to the other classes of connections. Rich club connections (purely selected on basis of topological properties of their connecting nodes) spanned significantly longer physical distances than connections spanning between peripheral nodes, involved a large proportion of the total macroscale wiring cost, were most often intermodular of character, and involved significantly more bidirectional projections than pathways linking peripheral nodes. Notably, the class of rich club connections showed significantly higher connectivity strength than the class of feeder and local projections, an effect not just driven by strong short-range connections, as also in particular the subclass of long-range (e.g. top 10 % of longest connections) rich club connections revealed significantly stronger connections than local connections. Further categorizing the edges of the network on basis of whether they spanned between nodes of the same or spanned between nodes of different modules, revealed a strong contribution of rich club edges to the class of intermodular edges, underscoring the rich club’s role in intermodular communication (Zamora-Lopez et al. 2010; van den Heuvel and Sporns 2013b).

Second, extending previously reported findings on global network features of mammalian neural networks, our findings show evidence of a non-uniform distribution of connectivity strength across the rat brain network. Subclasses of edges based on projection strength are found to show different topological properties, with edges of low strength (the class of weak connections) showing predominantly low levels of clustering, low community structure and to form relatively short communication paths. In contrast, high strength connections (the class of strong and very strong connections) showed high levels of clustering, strong community formation and relatively large subgraph paths. In addition, the weakest class of connections was found to have a relative high impact on (binary measured) global communication paths together with a relative low impact on global network clustering, suggesting a relative widespread and relative unique distribution of these connections across the network (Fig. 8). Speculating on these findings, such a distribution suggest a potential random organization of weaker neural pathways and a relatively ordered organization of stronger connections in the mammalian brain (de Lange et al. 2014). These findings coincide with observations from developmental studies, noting widespread—possibly somewhat random- termination zones of long-range corpus callosal tracts at birth (day 4, as observed in the visual system of the cat), followed by a relatively short period (~days to weeks) in which rapid spatial specialization of cortico-cortical tracts and their termination zones occurs (Huttenlocher and Dabholkar 1997). Such an initial overgrowth of macroscopic connectivity in the pre-term developing brain is supported by empirical observations in the young rhesus monkey, revealing the highest axonal connection count of corpus callosal fibers at birth, followed by a decrease (up to 70 %) in axonal number in the first postnatal weeks (LaMantia and Rakic 1994). In context of these developmental findings, a speculative hypothesis on the basis our current observations of weak macroscale projections in the adult mammalian brain showing a widespread and relatively random organization may include the notion that the class of weak connections are somewhat ‘left-over’ –and thus potentially non- or less functional– pathways from a macroscale pruning period during brain development (van den Heuvel et al. 2014; Collin and van den Heuvel 2013). Following this hypothesis, potentially the strongest connections, as observed in the rat brain, are the pathways that have been subject to strong activation and activity during later development, strengthening their functional role in the total system and potentially leading to stronger anatomical pathways in the adult brain.

With their central embedding in brain networks, neural rich club hub nodes have been suggested to play an important role in shaping and routing global processes in neural systems (van den Heuvel et al. 2012; Zamora-Lopez et al. 2010). Their dense level of mutual connectivity has lead to the hypothesis that high degree regions do not work in isolation, but rather form a central anatomical infrastructure for neural communication and information integration, and thus potentially form an anatomical substrate of a neural ‘global workspace’ (Dehaene et al. 1998; Harriger et al. 2012; van den Heuvel and Sporns 2013a). Also in the current study of the rat connectome the neural rich club is found to stand out as an ideal candidate for such a central backbone for global integrative neural processes.

In addition, extending observations of hub and rich club formation in mammalian cortical brain networks (Zamora-Lopez et al. 2009; de Reus and van den Heuvel 2013; Sporns et al. 2007; Harriger et al. 2012; van den Heuvel and de Reus 2014), the rat connectome dataset now also includes information on anatomical connectivity of deeper gray matter structures, revealing an above average number of connections of hippocampal (CA1, centrality score of 3) and amygdala nuclei (LA, BMAp, centrality score of 3; BLAp, centrality score of 4). These findings provide tract-based support for preliminary observations of rich club formation in the human brain based on in vivo diffusion imaging data, suggesting a high number of macroscale connections of subcortical structures such as the amygdala, hippocampus and thalamus. A participation of subcortical regions in a high degree club of regions is of course consistent with numerous reports of these regions to form critical brain areas involved in memory and global information relaying.

As mentioned, imaging derived estimates of aspects related to ‘cost of wiring’ in human brain networks have suggested that hub regions and their connections may form a high cost neural structure, involving a large proportion of long-range projections, as well as pathways of high connectivity strength, high white matter volume, and high levels of microstructure (van den Heuvel et al. 2012; Collin et al. 2013). These observations have primarily been made on the basis of in vivo imaging-based markers which can at best, as noted (see for example for review Jones 2008; Johansen-Berg and Rushworth 2009; Jbabdi and Johansen-Berg 2011) only provide a crude estimate of the connectivity strength of white matter pathways. The current tract tracing based findings in the rat connectome are however in clear support of these earlier diffusion based observations: white matter pathways between rich club regions are again found to display on average a significantly higher connectivity strength as compared to other white matter pathways, to be most often bidirectional, and to span longer physical distances. Our findings thus provide important tract tracing validation of earlier neuroimaging-based observations, suggesting that neural hubs and their connections form a high cost feature of brain architecture (van den Heuvel et al. 2012; Bullmore and Sporns 2012). A high cost character of neural hubs and their connections may lead to the hypothesis of a –to some extend- potential concentration of connectivity to central regions in neural systems. In the rat dataset, the number of low versus high strength connections of nodes are observed to significantly correlate with the total number of nodal connections, with high degree regions showing (on average) more high-strength connections than low-strength connections. Across all individual connections the connectivity strength of pathways is observed to increase with the combined degree of the target and source regions of a connection, suggesting that pathways that span between higher degree regions also have a higher probability of displaying stronger axonal pathways. These findings thus suggest that neural hubs and their connections are not only ‘rich’ in term of their number of pathways, but also in terms of above average projection strength of their connections.

Studies have reported on an efficient communication architecture of the neural systems of several mammalian species, including the mouse, cat, macaque, chimpanzee and human brain. While general caution is warranted when assuming homology across species (Sereno and Tootell 2005), studies have consistently reported on a consistent modular structure of mammalian neural systems (e.g. Hagmann et al. 2008; Stephan et al. 2000; Salvador et al. 2005; Damoiseaux et al. 2006; van den Heuvel et al. 2008a), as well as reported on the formation of neural hubs with relative large overlap in both their spatial and their topological position in the overall network (e.g. Sporns et al. 2007; Bullmore and Sporns 2012; Tomasi and Volkow 2010). Furthermore, comparing macroscale neural networks across mammalian species has revealed considerable overlap in their global architecture (e.g.Goulas et al. 2014; van den Heuvel and Sporns 2013b) which, together with the current observations, tends to suggest the existence of a set of biological rules that influence the global organization of macroscale wiring of neural systems and potentially a common mode of functioning of neural systems across (mammalian) species. However, the apparent overlap in global wiring pattern also brings up the question of potential differences in more fine-grained aspects of brain connectivity. Besides overlap, studies have noted across-species differences in the connectivity profiles of specific regions (e.g. Li et al. 2013; Neubert et al. 2014), showing for example clear differences in the connectivity fingerprints of frontal regions of the human and macaque brain. Future studies expanding such investigations, focusing on potential differences in the global wiring architecture of neural systems between human and other species are of particular interest.

Some points should be taken into consideration when interpreting the findings of this study. First, the BAMS rat brain dataset -similar to other pioneering endeavors to map the connectomes of the cat, macaque, mouse brain and nervous system of the C Elegans- comprises the reconstruction of a connectome based on the aggregation of data from a large number injection experiments and does not involve the reconstruction of a macroscale connectome of a single specimen (van den Heuvel and de Reus 2014; Scannell et al. 1995; Stephan et al. 2001). The examined connectome dataset thus does not provide information on individual variation of brain wiring. In addition, the BAMS-II database does not yet include information on connectivity (or absence of connectivity) on all region-to-region pairs, and database updates incorporating information on more and more connection pairs are thus regularly made (Bota et al. 2012; Bota and Swanson 2007). Second, it is important to note that the examined rat connectome dataset (that is, how we used it) only represents information on intra-hemispheric connectivity (with tract-tracing data across the left and right hemisphere combined), and the absence of information on corpus callosal tracts is likely to influence the graph theoretical analysis. Callosal tracts are known to involve a large proportion of all white matter tracts (Schamahmann and Pandya 2006) and to play an important role in interhemispheric functional connectivity and communication (e.g. (LaMantia and Rakic 1990; Lowe et al. 2008; Pandya et al. 1971; Wahl et al. 2007; Verstraete et al. 2011; van den Heuvel and Hulshoff Pol 2010b). Notably, a recent tract tracing study in the adult mouse brain revealed an intriguingly high level of inter-hemispheric connectivity as well as a potential non-trivial organization of these projections (Oh et al. 2014), highlighting an important role for these connections in global connectome structure.

Further corroborating on emerging evidence of an efficient communication architecture of neural systems, our findings suggest a non-uniform distribution of the connectivity strength across white matter pathways in neural systems, with weak connections showing a predominantly random-like organization and strong connections showing high levels of local organization. Anatomical connections between high degree hub nodes are observed to show on average a higher connectivity strength as compared to other types of connection pathways, further extending the notion of neural hubs to form a high cost, high capacity infrastructure in the mammalian brain. Future studies examining whether and if so to what extent weak and strong pathways differ in their macroscale neuroarchitectonics might provide important insights into the hypothesized disproportionally high vulnerability of high degree regions and their connections in disease processes (van den Heuvel and Sporns 2013b; Crossley et al. 2013, 2014).

## Electronic supplementary material

Below is the link to the electronic supplementary material.

Supplementary material 1 (PDF 109 kb)
