# Supplementary material for: Topological organization of connectivity strength in the rat connectome
Source: Brain Struct Funct. 2015 Feb 20;221(3):1719–36. doi: 10.1007/s00429-015-0999-6 (PMC4819781; doi:10.1007/s00429-015-0999-6)
Supplement: Supplementary file 1 — Supplementary material 1 (PDF 109 kb) [file 429_2015_999_MOESM1_ESM.pdf]

Supplemental Table S1

Number	Region abbreviation	Region	Included in matrix
1	MOp	primary motor cortex	•
2	MOs	secondary motor cortex	•
3	SSp	primary somatosensory cortex	•
4	SSs	secondary somatosensory cortex	•
5	VISC	visceral area	•
6	ILA	infralimbic area	•
7	GU	gustatory area / dysgranular insular area	•
8	MOB	main olfactory bulb	•
9	AOB	accessory olfactory bulb	
10	AON	anterior olfactory nucleus	•
11	TTd	dorsal tenia tecta	•
12	TTv	ventral tenia tecta	•
13	PIR	piriform area	•
14	TR	postpiriform transition area	•
15	PAA	piriform amygdalar area	•
16	NLOT	nucleus of the lateral olfactory tract	•
17	COAa	anterior cortical amygdalar nucleus	•
18	COApl	posterior lateral cortical amygdalar nucleus	•
19	COApm	posterior medial cortical amygdalar nucleus	•
20	AUDp	primary auditory cortex	•
21	AUDd	dorsal auditory areas	•
22	AUDv	ventral auditory areas	•
23	VISlla	anterior laterolateral visual area	•
24	VISal	anterolateral visual area	•
25	VISam	anteromedial visual area	•

Number	Region abbreviation	Region	Included in matrix
26	VISli	intermediolateral visual area	•
27	VISll	laterolateral visual area	•
28	VISlm	mediolateral visual area	•
29	VISpl	posterolateral visual area	•
30	VISp	posterior lateral area	•
31	VISrl	rostrolateral visual area	•
32	ACAd	dorsal part of the anterior cingulate area	•
33	ACAv	ventral part of the anterior cingulte area	•
34	PL	prelimbic area	•
35	ORBl	lateral orbital area	•
36	ORBm	medial orbital area	•
37	ORBv	ventral orbital area	•
38	ORBvl	ventrolateral orbital area	•
39	AId	dorsal agranular insular cortex	•
40	AIv	ventral agranular insular cortex	•
41	AIp	posterior agranular insular cortex	•
42	RSPd	dorsal retrosplenial cortex	•
43	RSPagl	retrosplenial agranular area	•
44	RSPv	retroplenial granular area	•
45	PTLp	posterior parietal association areas	•
46	TEa	temporal association areas	•
47	ECT	ectorhinal area	•
48	PERI	perirhinal area	•
49	ENTl	entorhinal area, lateral part	•
50	ENTm	entorhinal area, medial part, dorsal zone	•
51	ENTmv	entorhinal area, medial part, ventral zone	•

Number	Region abbreviation	Region	Included in matrix
52	PRE	presubiculum	•
53	POST	postsubiculum	•
54	PAR	parasubiculum	•
55	SUBd	subiculum, dorsal part	•
56	SUBv	subiculum, ventral part	•
57	CA1	Hippocampal region CA1	•
58	CA2	Hippocampal region CA2	•
59	CA3	Hippocampal region CA3	•
60	DG	Hippocampal region, dentate gyrus	
61	IG	Hippocampal region, Induseum Griseum	
62	FC	Hippocampal region, fasciola cinerea	•
63	CLA	Claustrum	•
64	EPd	Endopiriform nucleus, dorsal part	•
65	EPv	Endopiriform nucleus, ventral part	•
66	LA	Lateral amygdalar nucleus	•
67	BLAa	Basolateral amygdalar nucleus, anterior part	•
68	BLAp	Basolateral amygdalar nucleus, posterior part	•
69	BMAa	Basomedial amygdalar nucleus, anterior part	•
70	BMAp	Basomedial amygdalar nucleus, posterior part	•
71	PA	Posterior amygdalar nucleus	•
